# How Followership Boosts Creative Performance as Mediated by Work Autonomy and Creative Self-Efficacy in Higher Education Administrative Jobs

**DOI:** 10.3389/fpsyg.2022.853311

**Published:** 2022-05-31

**Authors:** Hua-Ling Chiang, Yung-Chih Lien, An-Pan Lin, Ya-Ting Chuang

**Affiliations:** ^1^Department of International Business, College of Management, National Taiwan University, Taipei, Taiwan; ^2^Department of Civic Education and Leadership, National Taiwan Normal University, Taipei, Taiwan; ^3^Office of Administrative Affairs, National Taiwan Normal University, Taipei, Taiwan

**Keywords:** effective followership, work autonomy, creative self-efficacy, creative performance, trait activation theory

## Abstract

Followership is an important but understudied domain. This study adopted a follower-centric perspective to examine the internal process by which followership affects creative performance via work autonomy and creative self-efficacy. The study employed a 3-wave survey of 341 employees of a Taiwanese university to achieve the research purpose. This study showed that effective followership (Time 1) is positively associated with employees’ work autonomy (Time 1) and creative self-efficacy (Time 2). Work autonomy and creative self-efficacy mediate the relationship between effective followership and creative performance (Time 3). This study’s empirical findings provide an improved way of measuring followership and broaden our understanding of how followership triggers intrinsic motivation to facilitate creative performance.

## Introduction

The followership-centric perspective has gradually attracted attention in recent years (e.g., Peterson et al., 2020; Armstrong, 2021). Scholars have found that teaching followership in managerial courses is necessary (Northouse, 2018; Jenkins and Spranger, 2020) because followership development empowers long-term success for groups (Armstrong, 2021). Followers directly contribute to approximately 80% of organizational success, whereas leaders may contribute to only 20% of such success (Kelley, 1992). Kelley (2008) pointed out that traditionally, enterprises and scholars adopted a leadership-centric view and were less inclined to adopt the followership-centric view. Instead of merely following procedures about how to act, good followership requires reflexivity, thoughtfulness, and a recognition of one’s shortcomings. That is, followers may actively submit their creative ideas and inspirational example to the group (Alvesson and Blom, 2019). Scholars have continuously called for further research on and awareness of followership development (Kelley, 2008; Uhl-Bien et al., 2014; Bufalino, 2018). There are 195 empirical studies that explore the issue of leadership and creativity in the ProQuest, PsycInfo, EBSCO, and ISI Web of Science databases (Hughes et al., 2018). In contrast, few studies have examined the type of followership related to creative job performance; thus, the present study explored how followership can boost employees’ creative performance in their jobs.

According to Miner (1993), role motivation theory states that in an organizational hierarchy, different positions have different job roles that, together with employees’ own motivational requirements, allow for their effective performance (Stollberger et al., 2019). Considering that motivation is a force that triggers and helps sustain task-related focus and effort (Pinder, 1998), the development of creativity in an organization from the social cognition perspective (Bandura, 1991) captures the creative behaviors of colleagues, which can then motivate other employees’ creativity (Huang et al., 2016). Among the various motivational attributes, creative self-efficacy (CSE) has a unique ability to influence employees’ creativity. Tierney and Farmer (2011) found that as CSE is a domain-specific form of efficacy, it can predict creativity. In addition, due to the outbreak of the new coronavirus, a large number of public and private organizations practice a work from home policy. Correspondingly, employees are allowed a higher level of work autonomy. Autonomy refers to employees largely self-directing themselves in their work; many highly qualified employees who are motivated by interesting work have the ability to work on their own and require only occasional support from their colleagues (Alvesson and Blom, 2019). Importantly, followership can give members a “feeling” of participation and promise an alternative to enhance job performance, even under pressure (Plachy and Smunt, 2021). Overall, the relationship between motivational attributes and creative performance has not been extensively studied; thus, the present study focused on how employees’ followership can affect their CSE and work autonomy as reflected in their creative performance.

The specific contribution of this study is to adopt a followership-centric view to understand how effective followers facilitate creative performance via CSE and work autonomy, which addresses the deficiency of previous studies that particularly emphasized the leadership-centric perspective. In a method aspect, this study reviews Kelley’s followership instrument in the Taiwanese context and applies three time points to collect data.

## Theoretical Background

### Followership

Followership-centric approaches are distinct from leadership-centric approaches in which followers act as vassals in the leadership process (Uhl-Bien and Pillai, 2007; Uhl-Bien et al., 2014). A followership-centric approach can be distinguished from a role-based view and a constructionist view. The role-based view treats followership as a role and observes followers’ behaviors directly, whereas the constructionist view considers followership a social process that is associated with leadership (Uhl-Bien et al., 2014). To ensure that the group cannot be dominated by a single leader, followers work together to monitor and scrutinize the leader’s decisions. Followers help organizations achieve coordination with the regulating mechanisms of norms, social contracts and reputation. Such mechanisms could influence leaders and help organizations achieve their goals (Van Vugt, 2014).

Kelley (1992) identified five styles of followership by using two characters, namely, active engagement and independent critical thinking. The first style, passive followers, shows low levels of active engagement and independent critical thinking. Second, conformists, who are also referred to as obedient followers, also lack critical thinking but have high levels of initiative and motivation. The third style, alienated followers, tends to have low levels of engagement but can engage in independent and critical thinking. Fourth, pragmatists act in accordance with the time and the place, just go with the flow and carry their share. The fifth style, effective followers, shows high levels of active engagement and critical thinking. Travis (2015) study involving followership in Indiana hospital industries found that there was an association between the effective followership style that features higher levels of active engagement and critical thinking compared with the other four styles of followership. This finding indicates that effective followership can result in higher levels of team coordination. Accordingly, an effective followership style was used in this study to investigate employees who work at higher education institutions.

### Work Autonomy

Meaningful work was defined by Both-Nwabuwe et al. (2017) as individuals finding their work experience to be vital and valuable. Understanding precisely what makes work meaningful is crucial for autonomy (Martela and Pessi, 2018). That is, when an individual has a sense that they own their work and feel that they can perform it and it is truly of interest to them, the work is likely to feel personally meaningful to them (Martela et al., 2021). Autonomy refers to a sense of volition and internal locus of causality in individuals’ undertakings where individuals have ownership of their actions to undertake tasks that they feel are meaningful (Ryan and Deci, 2000; Chirkov et al., 2003). Karasek and Theorell (1990) argued that autonomy is a crucial feature of job design, as it allows employees to complete their work tasks at their own volition and according to their own steps, procedures, and modes (Spreitzer, 1995; Volmer et al., 2012). Both-Nwabuwe et al. (2019) found a positive relation between professional autonomy, which is found in areas of work such as nursing, and most dimensions of meaningful work. In addition, some employees gained more work autonomy because of flexible working patterns during the pandemic (Reisinger and Fetterer, 2021). Accordingly, the professional autonomy of employees who work in higher education institutes is the topic of interest in this study.

### Creative Self-Efficacy

Self-efficacy may influence one’s choice of activity, the amount of effort exerted, persistence, and ultimate attainment of a given outcome (Schunk and DiBenedetto, 2020). CSE, which Tierney and Farmer (2002) defined as an individual being self-confident about making innovative products, is a specific construct of self-efficacy, that is, one’s perceived ability to succeed in achieving particular tasks (Bandura, 1997). Beghetto and Karwowski (2017) pointed out that CSE forms one’s creative tendency, which is to make creative efforts to overcome creative challenges. When professionals can better master their field of expertise and the necessary skills, their CSE will be higher, which allows them to think of creative solutions (Capron Puozzo and Audrin, 2021). In line with this, the participants of this study were administrators who worked in Taiwan higher education, and their CSE was of interest in this study.

### Creative Performance

There is evidence of an intercorrelation between job performance and creativity factors (Ree et al., 2015). However, there is also evidence of the creative aspect of job performance (Cleveland et al., 2019). As a construct, creative performance (CP) includes the generation of novel and useful ideas related to the processes and procedures of one’s work (Oldham and Cumming, 1996). As jobs become increasingly complex and as unpredictability in organizations and their environments increases, Pulakos et al. (2000) suggest that it will become increasingly important for effective job performance to adapt to new demands and new circumstances. Employees’ CP will be needed to address the novel demands of their jobs (Cleveland et al., 2019). For example, in the times of the COVID-19 pandemic crisis, when organizations face unpredictable challenges, CP is crucial to sustaining competitive power (Tønnessen et al., 2021). In addition, during the COVID-19 pandemic, universities encounter complex change and need their employees to perform more creatively; thus, the question of how university employees can be more creative in their job performance to overcome complex challenges is a topic of interest in this study.

### Research Model and Hypotheses

#### Research Model

**FIGURE 1:**
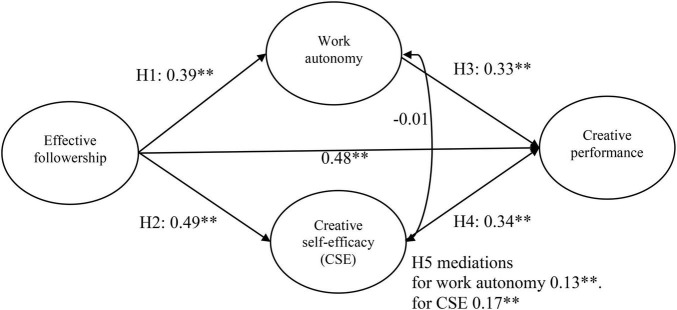
Model of the results of this study. The coefficients of the mediating results used H1(0.39)*H3(0.33) and H2(0.49)*H4(0.34); thus, CSE exhibited stronger indirect effects than work autonomy in the model. ^**^p < 0.01.

An important interaction between individuals’ internal traits and situations is emphasized by trait activation theory (TAT) (Tett and Burnett, 2003), according to which personality attributes are expressed within trait-relevant scenarios. It also explains that the latent personality traits in which workplace situations are aroused are critical (Tett and Burnett, 2003, p. 502). Work autonomy is one of the crucial organizational contextual factors that impacts creativity. However, individuals’ self-efficacy is the main determinant of accepting challenging tasks based on social cognitive theory (Bandura, 1982). Some studies have verified that CSE plays a mediating role in the relationship between leadership and creativity (Hughes et al., 2018). This issue takes into account CSE as a mediator that explains the mechanism of the relationship between followership and CP. Considering that personal characteristics play an essential role in CP (Amabile, 1996) and as a predictive key of work processes, they are likely to be a source that impacts employees’ potential triggers to exhibit their own self-efficacy and work autonomy to reflect CP; thus, the focus of this study is to explore the relationship between effective followership judgments and CP. More specifically, we are interested in how university administrators judge their followership and their CP mediated by their CSE and work autonomy. The research model is presented in Figure 1.

##### Hypotheses

Effective followers are usually competent workers who can collaborate well and are public supporters of organizational leadership (Howell and Mendez, 2008). When facing work problems, if effective followers have an idea of how to do something that will require more than just their own individual efforts, then they will have to communicate this proposal to others (Pietraszewski, 2020). In contrast, other types of followers can manage and coordinate their work by giving up their autonomy and following what they are told to do and how to do it (Van Vugt, 2017). Moreover, a previous study indicated that library workers are well-educated and tend to have a great deal of autonomy while working in organizations with effective followership (Martin, 2019). The mechanisms of followership can regulate employees to develop “if-then” algorithms (Bastardoz and Van Vugt, 2019). If employees who work in higher education institutes can be considered well-educated people, then to understand the correlates between effective followership and work autonomy in college administration, a hypothesis was proposed as follows:

**Hypothesis 1**: Effective followership is positively related to employees’ work autonomy.

CSE is defined as the confidence that one possesses in one’s individual capacity to generate creative results (Tierney and Farmer, 2002). In workplaces, all types of behaviors ranging from creditable to unseemly can be found. Some employees pursue the organizational goals and their own legitimate goals (Ones, 2002). A previous study suggested that followers sometimes focus on the desirability of actions and outcomes (Berson et al., 2015). Moreover, training employees to be critical thinkers can promote their CSE to the degree that they believe that they have the ability to engage in creative processes (Tantawy et al., 2021). Based on these earlier studies, we adapted the effective follower role with critical thinking and creative engagement that reflect their CSE; thus, to understand how effective followership can predict CSE, a hypothesis was proposed as follows:

**Hypothesis 2**: Effective followership is positively related to employees’ CSE.

Creative work tends to involve complex and ambiguous tasks and requires discretion and autonomy on the part of employees (Mumford et al., 2002). The advantage of autonomy is that qualified employees who need less supervision can make quick decisions and concentrate on their work and results (Alvesson and Blom, 2019). Professional autonomy has been defined as employees’ perceived freedom in their jobs, where freedom means that they can work without fear, they are not overly restricted by the rules of the organization, and they do not have to obtain consent, obey orders, or request permission to do their work (Both-Nwabuwe et al., 2020). As such, there is the possibility that they may do the wrong things in such a context with minimal managerial control, and this guides employees in their creative behavior (Alvesson and Blom, 2019). That is, knowledge work in complex tasks often calls for professional autonomy, with autonomy contributing to workplace creativity (Sia and Appu, 2015). Accordingly, to understand how university administrators’ work autonomy is related to their CP, a hypothesis was suggested as follows:

**Hypothesis 3**: Work autonomy is positively related to CP.

Regarding the role that CSE may play in CP, according to Bandura (1997), creativity requires protracted and unremitting efforts, which, in turn, require strong self-efficacy to continue with one’s creative efforts. Tierney and Farmer (2002) also considered CSE to be a major premise to creative effort and essential for continuing one’s pursuit of CP, especially in the face of obstacles (He and Wong, 2021). Farmer and Tierney (2017) described CSE as a particular type of self-efficacy that refers to one’s perception (“the self”) of being capable of achieving creative outcomes. That is, people who have high levels of CSE are usually better at perceiving opportunities rather than obstacles when they are faced with challenging situations (Newman et al., 2018). They also have a greater tendency to develop and implement new ideas to increase their creative job performance (Liao et al., 2021). Accordingly, to understand how CSE can predict the creative job performance of higher education administrators, a hypothesis was proposed as follows:

**Hypothesis 4**: CSE is positively related to CP.

Based on self-determination theory (Deci et al., 2017; Ryan and Deci, 2017), the basic needs of autonomy and competence are the intrinsic motivations of human beings. In some studies, self-efficiency and competence are used interchangeably (Spreitzer, 1995; Samson and Solmon, 2011; Rodgers et al., 2014). After integrating the hypotheses above, work autonomy and CSE were assumed to be mediators between effective followership and CP. Effective followers stimulate themselves through intrinsic motivation to perform creatively and obtain satisfaction in this process. Therefore, the following hypothesis was proposed:

**Hypothesis 5**: Work autonomy and CSE mediate the effects of followership on CP.

## Methods

### Procedure and Participants

Data were collected from the employees of a higher education institute in Taiwan at three different time points. Adopting a temporal design is useful to identify the theoretical model and allow for an operationalization of statistical analysis that matches the model while simultaneously avoiding the inclusion of irrelevant or meaningless scientific questions and giving explicit answers to the research questions (Collins, 2006). First, 356 employees in the administrative units were recruited and provided their informed consent to participate. To investigate longitudinal data, all participants were required to use a unique serial number as their research ID when responding to the survey to maintain anonymity. Random serial numbers were printed on each informed consent form. First, 356 employees working in a variety of administrative units provided their informed consent. The first survey link was distributed alongside the informed consent form, which was presented on paper, and the measurement included items related to the employees’ demographic variables, self-rated followership, and work autonomy. We separated the collection of participants’ e-mail addresses and sent a second survey webpage with a corresponding link 2 weeks after the first survey was distributed. The second survey measurement included creative self-efficiency. The third survey was sent 2 weeks after the second survey. The third survey measurement was CP.

In total, 341 employees responded to the surveys (96% response rate), which included incomplete surveys at either Times 2 or 3. Among the employees, 25.5% were male, and 74.5% were female; the average age was 37.96 years (*SD* = 9.42), and the average organizational tenure was 7.59 years (*SD* = 9.70). Approximately 55.4% had a bachelor’s degree, and 39% had a graduate degree.

### Measurement

#### Effective Followership

Kelley (1992) followership measurement in a Chinese context is questioned. This study recruited 147 participants from the administrative departments of two higher education institutes in Taiwan (23.8% were males, 76.2% were females; mean age = 42.05, *SD* = 9.73; average organizational tenure = 10.36 years, *SD* = 10.06; 42.2% held a bachelor’s degree, and 53.8% held a graduate degree) through either mail or direct contact. Some participants (approximately 58.9%) joined Study 2. This study adopted the cross-validation approach (see Sonnentag and Fritz, 2007; Gatti et al., 2014) and randomly divided the collected data into two subsamples. However, we initially found it difficult to obtain a perfect model fit through two random split samples. Thus, this study recruited four experts (including two Ph.D. candidates of education and international management, one psychological doctor, and one testing expert) to review Kelley’s 20-item questionnaire, and they scored each item to quantify their subjective opinions regarding whether the items matched the intention of Kelley’s two dimensions. The experts scored each item from 0 to 10 (0 = very poor; 10 = very suitable), and an item with an average score lower than 5 was considered unsuitable and was deleted. During this stage, 13 items were retained (see Supplementary Appendix).

Then, we adopted the cross-validation approach again; the first subsample is identified as a calibration sample (*n* = 72) with the best-fitting model, and the second subsample is identified as a cross-validation sample (*n* = 75) to validate this model. To calibrate the sample data, we used SPSS 22 software to conduct an exploratory factor analysis with maximum-likelihood extraction and varimax rotation, and we assume that Kelley’s two-dimensional followership concept is feasible; thus, we set the number of factors as 2. If the factor loading was lower than 0.5 and belonged to an unexpected dimension, then these unsuitable items were deleted. Then, we used the cross-validation sample to further examine the factor structure by conducting a confirmatory factor analysis (CFA) with Mplus 8.4. We included retained items in the calculation and obtained a reasonable model fit [χ^2^ = 68.03, *df* = 34, *comparative fit index (CFI)* = 0.94, *Tucker-Lewis index (TLI)* = 0.92, *root mean square error of approximation (RMSEA)* = 0.11, *standardized root mean square residual (SRMR)* = 0.05]. The retained items were used in the analysis of the main study. The alpha coefficient of all 147 samples was 0.91 for active engagement and 0.85 for critical thinking.

Items were adapted from Kelley (1992) followership scale and were assessed by the reviewed seven-item effective followership style questionnaire. The employees were required to respond to items on a 7-point Likert scale (0 = never; 6 = always). Example items include “When starting a new job or assignment, do you promptly build a record of successes that are important to the organization and its leaders?” and “Do you independently think of and champion new ideas that will contribute significantly to the organization’s goals?” The Cronbach’s alpha was 0.97 in this study.

#### Work Autonomy

Work autonomy was measured by using three items from Spreitzer (1995) scale of self-determination. The employees were asked to answer items on a 5-point Likert scale (1 = completely disagree; 5 = completely agree). A sample item is “I have significant autonomy in determining how I do my job.” The Cronbach’s alpha for work autonomy was 0.90 in this study.

#### Creative Self-Efficacy

Creative self-efficacy was measured with four items from Tierney and Farmer (2002) CSE scale. The employees rated the extent to which they had experienced a certain state “in the last month” on a 5-point Likert scale (1 = completely disagree; 5 = completely agree). A sample item is “I am good at finding creative ways to solve problems.” The Cronbach’s alpha for CSE in this study was 0.93.

#### Creative Performance

Creative performance was measured by using three items from Oldham and Cumming (1996) employee creativity measure. The subordinates rated the extent to which they had experienced a certain state “over the past 3 months” on a 7-point Likert scale (1 = completely disagree; 7 = completely agree). A sample item is “I am original and practical in my work.” The Cronbach’s alpha for CP in this study was 0.93.

## Results

### Model Fit Analysis

The analyses were conducted with Mplus 8.4 (Muthén and Muthén, 1998-2017) to test the hypothesized relations. We first conducted a CFA of effective followership (by adopting a higher-order factor with active engagement and critical thinking), work autonomy, CSE, and CP to verify that each variable was unique. We also conducted structural equation modeling (SEM) to assess the main and mediating effects, including the direct effect of effective followership on creative performance and the covariation of work autonomy and CSE; both CFA and the SEM showed acceptable model fit (χ*^2^* = 155.30, *df* = 111, *comparative fit index CFI* = 0.99, *TLI* = 0.99, *RMSEA* = 0.03, *SRMR* = 0.03). We provided the indirect associations and confidence intervals (CIs) to represent the mediating effect.

Table 1 displays the means, standard deviations, correlations, average variance extracted (AVE), and composite reliability (CR) of the study variables. The AVE for the constructs ranged from 0.54 to 0.96, i.e., above the threshold of 0.50, CR values ranged from 0.84 to 0.99, i.e., greater than 0.7 (Hair et al., 1998), and the square roots of the AVE values (AVEs) of each construct were greater than the squared correlation values, i.e., the diagonal values were greater than the off-diagonal values (Fornell and Larcker, 1981), representing the adequate discriminant validity of different constructs.

**TABLE 1:** Means, standard deviations, correlations, average variance extracted, and composite reliability of the study variables.

Variables	Mean	SD	1	2	3	4	AVE	CR
1. Effective followership	5.09	0.94	(0.73)				0.54	0.88
2. Work autonomy	3.86	0.71	0.52**	(0.80)			0.64	0.84
3. Creative self-efficacy	3.60	0.66	0.55**	0.30**	(0.98)		0.96	0.99
4. Creativity performance	5.04	1.03	0.57**	0.46**	0.54**	(0.96)	0.93	0.98

n = 277–341 observations; **p < 0.01; two-tailed tests. Correlations used the Pearson correlation coefficient to calculate the means of the variables. Values in brackets indicate the square-root values of the AVEs for each construct.

### Hypothesis Verification

Figure 1 illustrates the results of the analysis. The direct effect of effective followership on creative performance was found (γ = 0.48, *p* < 0.01). In support of Hypotheses 1 and 2, the results show that effective followership was positively related to work autonomy (γ = 0.40, *p* < 0.01) and CSE (γ = 0.49, *p* < 0.01).

Furthermore, work autonomy was positively related to CP (γ = 0.33, *p* < 0.01), and CSE was positively related to CP (γ = 0.34, *p* < 0.01), thus supporting Hypotheses 3 and 4.

Table 2 shows that CSE mediated the relationship between effective followership and CP (indirect effect = 0.17, 95% CI = [0.08 0.26]) and that work autonomy mediated the relationship between effective followership and CP (indirect effect = 0.13, 95% CI = [0.06 0.20]). The 95% CIs of both measures excluded zero. Through the coefficients of the indirect results, CSE revealed a stronger indirect effect than work autonomy in the relationship between effective followership and CP.

**TABLE 2:** Indirect effect and confidence intervals (CIs).

	Indirect effect	95% confidence interval	
		Lower	Upper
Effective followership → Work autonomy → Creative performance	0.13	0.06	0.20
Effective followership → Creative self-efficacy → Creative performance	0.17	0.08	0.26

## General Discussion

### Interpretation of Results

Trait activation theory personality traits are expressed as responses to situational cues that are relevant to the trait (Tett and Burnett, 2003). The current study therefore focused on how university administrators judge their followership and their CP as mediated by their CSE and work autonomy. The results indicate that there are positive relationships among all four constructs.

When facing work problems, if effective followers have an idea of how to do something that will require more than just their own individual efforts, then they will have to communicate this proposal to others (Pietraszewski, 2020). As employees who work in higher education institutes can be considered to be well-educated persons, how their effective followership is related to their work autonomy was particularly investigated in this study. In examining Hypothesis 1, the results indicated that effective followership is positively related to professional autonomy. This result is consistent with a previous study that implied that library workers are well-educated and have a great deal of autonomy. They therefore tend to work in organizations with effective followership (Martin, 2019).

Creative self-efficacy is defined as the confidence that one possesses in an individual capacity to generate creative results (Tierney and Farmer, 2002). Moreover, when professional employees are critical thinkers, they can engage in their work and promote their CSE (Tantawy et al., 2021). In the workplace, a wide range of behaviors from laudable to ethically contemptible can be found (Ones, 2002). Accordingly, to understand how effective followership can predict CSE in higher education institutes, Hypothesis 2 was positively verified.

An advantage of autonomy is that qualified employees who need less guidance or supervision can make quick decisions and can concentrate on their work and results (Alvesson and Blom, 2019). Professional autonomy means a perception of a high level of freedom in a job. With freedom, employees take actions without fear and without considering organizational rules (Both-Nwabuwe et al., 2020). Knowledge work in complex tasks often calls for professional autonomy, with autonomy contributing to workplace creativity (Sia and Appu, 2015). Accordingly, to understand how university administrators’ professional work autonomy is related to their CP, Hypothesis 3 was positively verified. That is, people act in creative ways in a context with minimal managerial control (Alvesson and Blom, 2019).

Creative self-efficacy as one type of self-efficacy refers to a person’s self-understanding that he or she can achieve creative outcomes (Farmer and Tierney, 2017). That is, individuals who have high levels of CSE are likely to be more able to perceive opportunities rather than obstacles and can persevere when faced with challenging situations (Newman et al., 2018). When they encounter difficulties in the process of developing and implementing new ideas, they are also likely to feel more capable of rolling with the punches and of creatively improving their job performance (Liao et al., 2021). Accordingly, to understand how CSE can predict the creative job performance of higher education administrators, Hypothesis 4 was positively verified. This result is consistent with previous studies and indicates that it is crucial to regard CSE as an antecedent to creative effort to continue pursuing CP, especially when faced with obstacles (Tierney and Farmer, 2002; He and Wong, 2021).

Choi (2004) noted the mediating role of CSE in the relationship between individual personality traits and CP. Creative followers typically have unique followership characteristics (Oldham and Cumming, 1996). Kelley (1992) pointed out that followers with independent thinking are innovative and creative. Environments can orient the developing person’s actions and interactions. If a job is meaningfully perceived and can be performed by a person, then the environment creates progressively more complex trajectories that exhibit continuity development of CP over time (Bronfenbrenner, 1999). Amabile (1996) also indicates that creativity is a key dynamic for the sustained growth of organizational productivity. Thus, examining the effect of followership on CP is meaningful and balances the prevailing focus on the effects of leadership on creativity in the literature (e.g., Gong et al., 2009; Williams et al., 2017). Previous research has found that the ability of people to accurately judge their own critical and creative thinking is important to accurately determine self-performance (e.g., Panadero et al., 2019). Environments can orient the developing person’s actions and interactions. For example, in the university environment, Kelley (1992) pointed out that followers with independent thinking are innovative and creative, and professors were found to claim that creative thinking and problem-solving abilities are key in both education and the workplace (Pesout and Nietfeld, 2021). Drawing on the person-process-context-time model (PPCT) (Bronfenbrenner, 1988) to understand how effective followership affects CP in higher education environments and to understand how the university environment affects employees who exhibit effective followership that affects CP, the role of CP was proposed and positively verified in this study. This finding is consistent with previous research that found that the ability of people to make accurate judgments of their own critical and creative thinking is important to accurately guide CP (e.g., Panadero et al., 2019).

### Theoretical Implications

This study adopted a followership-centric view to improve the understanding of followership and responded to scholars’ calls (Kelley, 2008; Uhl-Bien et al., 2014; Bufalino, 2018). This study reviewed Kelley’s followership questionnaire items to achieve qualitative justifiability. Then, we conducted a statistical analysis to take into account the methodological issues raised by researchers and to allow us to retain the feasible items. This approach allowed us to match Kelley’s two-dimensional conception of followership more precisely by achieving scale purification (Wieland et al., 2017).

Specifically, in this study, we examined two insufficiently studied areas in the followership literature. First, we examined followership’s internal motivation, which is the root of followers’ actions (Gagné and Deci, 2005) and facilitates followers’ ability to achieve work outcomes. Second, scholars emphasize that effective followership is creative and innovative (Kelley, 1992; Jaussi et al., 2008), but no study has yet revealed this phenomenon. In practice, an increasing number of organizations make good use of the creative performance of their employees as a means of keeping pace with a changing environment. This study employed the variables of CSE and CP to provide empirical evidence. The findings may balance the prevailing focus on leadership in the innovation literature.

### Practical Implications

This study empirically demonstrates that employees with better followership can motivate themselves to improve their CP. University administrative managers may adopt “effective follower-centric goal setting and review” (Armstrong, 2021), which allows both supervisors and employees to discuss personal career goals that align with the organizational vision and review them annually or quarterly to ensure that both employees and the organization keep improving.

Bjugstad et al. (2006) indicated that followers stimulate themselves and that their motivation is internal; a leader merely taps into this internal strength of followers. The research results reveal that university administrative managers should adopt a promotion focus to increase their employees’ work autonomy by giving them more freedom to make decisions and adapting information technology to reduce tedious tasks for employees to produce better CP.

Creativity plays a vital role that may facilitate work performance. In this research, we were interested in the importance of CSE and how CSE may improve through an intervention to foster CP. Employees could collect first-hand experiences to demonstrate the impact of working creatively in their jobs. Meanwhile, university managers inspire colleges’ CSE by encouraging a growth mindset. By working in a competitive and uncertain environment, universities need administrators to work with creativity to face the transition of the work environment, which causes employees to have more opportunities to explore the possibilities of creative work. The results of this study can contribute to enhancing administrators’ CSE in working creatively.

### Limitations and Future Studies

With respect to Kelley (1992) original survey, this study adopted two main constructs in its research design; however, many items did not undergo content and statistical validation. It has been argued that other factors may exist in Kelley’s original measurement (Blanchard et al., 2009). This study suggests that future research should rethink followership constructs to improve the questionnaire.

This study was conducted in a higher education institute. As a result, occupational factors may affect the results. Furthermore, the cultural background of the study was Taiwan, which is a more collectivistic culture than Europe and the United States that tend to be more individualistic cultures. Future studies should recruit participants from various occupations and regions to enrich the validity of the study outcomes. Our findings can inspire further research to use a temporal design to conduct replication studies but also to employ all constructs of the study measuring each time point via cross-lagged panel models (Hamaker et al., 2015) to estimate the directional effects among variables over time.

## Conclusion

This paper studies this research topic, constructs a model based on TAT, cognitive theory, and self-determination theory and then analyzes it. A follower who combines high levels of active engagement and independent critical thinking has marked effective followership traits. Moreover, these two traits are triggered in workplaces with higher work autonomy. Furthermore, when the follower believes in his or her own ability to achieve innovative outcomes, he or she can show better CP. Accordingly, employees with effective followership can motivate themselves based on their need for autonomous work, which results in CSE that leads to positive work outcomes in their CP.

Organizations are made up of a majority of followers and a minority of leaders. Correspondingly, when more insight into followership is attained, competitive advantages can also be gained in the context of organizational governance.

## Data Availability Statement

The original contributions presented in the study are included in the article/Supplementary Material, further inquiries can be directed to the corresponding author.

## Author Contributions

H-LC: original draft and data collection. Y-CL: review and editing. A-PL: data collection and review and editing. Y-TC: data collection and data analysis. All authors contributed to the article and approved the submitted version.

## Conflict of Interest

The authors declare that the research was conducted in the absence of any commercial or financial relationships that could be construed as a potential conflict of interest.

## Publisher’s Note

All claims expressed in this article are solely those of the authors and do not necessarily represent those of their affiliated organizations, or those of the publisher, the editors and the reviewers. Any product that may be evaluated in this article, or claim that may be made by its manufacturer, is not guaranteed or endorsed by the publisher.
