# Supplementary material for: How Followership Boosts Creative Performance as Mediated by Work Autonomy and Creative Self-Efficacy in Higher Education Administrative Jobs
Source: Front Psychol. 2022 May 31;13:853311. doi: 10.3389/fpsyg.2022.853311 (PMC9194574; doi:10.3389/fpsyg.2022.853311)
Supplement: Supplementary file 1 [file Table_1.pdf]

Appendix

(1) Expert Scoring: An average score lower than 5 (inclusive) was considered unsuitable and marked, and the experts' comments are briefly described below.

Item	Original item from Kelley's measurement	Average score
AE1	(No. 2) Are your personal goals aligned with your student organization's priority goals?	5.00
AE2	(No. 3) Are you highly committed to and energized by your involvement and organization, giving them your best ideas and performance?	8.75
AE3	(No. 4) Does your enthusiasm also spread to and energize your peers?	3.75
AE4	(No. 6) Do you actively develop a distinctive competence in critical activities such that you become more valuable to the organization and its leaders?	7.25
AE5	(No. 7) When starting a new job or assignment, do you promptly build a record of successes that are important to the organization and its leaders?	8.00
AE6	(No. 8) Can the leader of your organization give you a difficult assignment without the benefit of much supervision knowing you will meet your deadline with high-quality work?	9.50
AE7	(No. 9) Do you take the initiative to seek out and successfully complete assignments that go above and beyond your role?	5.50
AE8	(No. 10) When you are not the leader of a project, do you still contribute at a high level, often doing more than your share?	6.25
AE9	(No. 13) Do you help your peers, making them look good, even when you don't get any credit?	5.00
AE10	(No. 15) Do you understand the leader's needs, goals, and constraints and work hard to meet them?	3.75
CT1	(No. 1) Does your involvement help you fulfill some societal goal or personal dream that is important to you?	3.50
CT2	(No. 5) Instead of waiting for or merely accepting what the leader tells you, do you personally identify which organizational activities are the most critical for achieving the organization's priority goals?	5.75
CT3	(No. 11) Do you independently think of and champion new ideas that will significantly contribute to the organization's goals?	8.75
CT4	(No. 12) Do you try to solve tough problems (technical, organizational, etc.) rather than look to the leader to do it for you?	8.25
CT5	(No. 14) Do you help the leader or organization see both the upside potential and downside risks of ideas or plans, playing the devil's advocate if needed?	6.75
CT6	(No. 16) Do you actively and honestly own up to your strengths and weaknesses rather than delay evaluation?	5.50
CT7	(No. 17) Do you make a habit of internally questioning the wisdom of the leader's decision rather than just doing what you are told?	5.75
CT8	(No. 18) When the leader asks you to do something that contradicts your preferences, do you say "no" rather than "yes?"	3.25
CT9	(No. 19) Do you follow your own ethical standards rather than the leader's or the group's standards?	6.25
CT10	(No. 20) Do you assert your views regarding important issues even though it might result in conflict with your group or leader?	4.50

(2) **Factor loading analysis of the sample calibration:** Based on the exploratory factor analysis results (maximum-likelihood extraction and varimax rotation, *n* =72), we deleted items that had a very high cross-loading with another item (CT 2 and CT6) or had low loadings (below 0.5) on the intended factor (AE7, AE8, CT7, and CT9). Therefore, AE2, AE4, AE5, AE6, CT3, CT4, and CT5 were retained.

**Item**	**Factor loading**	
	**Active engagement**	**Critical thinking**
AE2	0.794	0.285
AE4	0.865	0.289
AE5	0.697	0.463
AE6	0.857	0.252
AE7	0.059	0.229
AE8	0.198	0.666
CT2	0.822	0.320
CT3	0.409	0.827
CT4	0.457	0.717
CT5	0.373	0.563
CT6	0.644	0.421
CT7	0.192	0.186
CT9	0.392	0.252

(3) Goodness of Fit Statistics for the 7 Items Ultimately Retained

	χ2	*df*	*CFI*	*TLI*	*RMSEA*	*SRMR*
Calibration sample (n=72)						
One-factor model	63.76	14	0.88	0.80	0.22	0.07
Two-factor model	23.71	13	0.97	0.95	0.11	0.03
Cross-validation sample (n=75)						
One-factor model	75.79	14	0.81	0.72	0.24	0.09
Two-factor model	19.28	13	0.98	0.97	0.08	0.04

Items Ultimately Retained

- Active engagement: AE2, AE4, AE5, and AE6.
- Critical thinking: CT3, CT4, and CT5.
